# Evaluation of Reference Genes in the Polyploid Complex *Dianthus broteri* (Caryophyllaceae) Using qPCR

**DOI:** 10.3390/plants11040518

**Published:** 2022-02-14

**Authors:** Alba Rodríguez-Parra, Jesús Picazo-Aragonés, Francisco Balao

**Affiliations:** Departament of Plant Biology and Ecology, Faculty of Biology, University of Seville, Apdo. 1095, 41080 Seville, Spain; albarparra@gmail.com (A.R.-P.); fbalao@us.es (F.B.)

**Keywords:** reference genes, qPCR, *Dianthus broteri*, polyploidy

## Abstract

*Dianthus broteri* is an endemic complex which is considered the largest polyploid series within the Dianthus genus. This polyploid species involves four cytotypes (2×, 4×, 6× and 12×) with spatial and ecological segregation. The study of gene expression in polyploid species must be very rigorous because of the effects of duplications on gene regulation. In these cases, real-time polymerase chain reaction (qPCR) is the most appropriate technique for determining the gene expression profile because of its high sensitivity. The relative quantification strategy using qPCR requires genes with stable expression, known as reference genes, for normalization. In this work, we evaluated the stability of 13 candidate genes to be considered reference genes in leaf and petal tissues in *Dianthus broteri*. Several statistical analyses were used to determine the most stable candidate genes: Bayesian analysis, network analysis based on equivalence tests, geNorm and BestKeeper algorithms. In the leaf tissue, the most stable candidate genes were TIP41, TIF5A, PP2A and SAMDC. Similarly, the most adequate reference genes were H3.1, TIP41, TIF5A and ACT7 in the petal tissue. Therefore, we suggest that the best reference genes to compare different ploidy levels for both tissues in *D. broteri* are TIP41 and TIF5A.

## 1. Introduction

Gene expression analysis is a powerful strategy to examine the transcriptional profile of biological systems [1]. The quantitative polymerase chain reaction (qPCR) is one of the most appropriate techniques to measure the levels of gene expression through mRNA estimation, surpassing other methods such as DNA chip or Northern Blot [2,3]. Its main advantage is high sensitivity detection even for less abundant transcripts, high efficiency, specificity and simplicity [4].

There are two general approaches to analyze qPCR results, absolute and relative quantification. For the first one, a specific calibration curve with known concentrations is needed for each gene of interest. This strategy entails increased cost and time of analysis due to the need for identical amplification efficiency for both, the target gene in the biological sample and the synthetic standard. By contrast, the relative quantification does not require a calibration curve. In this strategy, the expression of the target genes is normalized using reference genes, also referred to as housekeeping genes, which are constitutively expressed and remain stable due to their implication in basic cellular processes [3,5]. This second approach seems easier than absolute quantification although reference genes need to meet some requirements [6]. The sensitivity of the qPCR analysis is affected by various factors such as RNA stability and quality, cDNA synthesis and quantity, the efficiency of the primers and the use of a suitable reference gene [7,8]. An ideal reference gene should have an immutable expression across different tissues and developmental stages, and their expression level should not be impacted by environmental conditions. Furthermore, reference candidates cannot be pseudogenes and their expression level must be in an accurate range, 15 < Cq < 30 [9]. Since there are no universally appropriate genes with invariant expression, it is essential to appraise the expression level of candidate reference genes with care for every specific experimental system [10].

While there are reference genes that have been recorded to be efficient over a large range of environmental conditions or plant tissues, there are hardly any studies focused on reference genes validation among different ploidy levels [10,11]. Polyploidy, the condition in which the cells of an organism have more than two paired sets of chromosomes (i.e., whole-genome duplication; WGD), takes place across many different taxonomic groups. Genomic data suggest that polyploidy has been one of the most important mechanisms in plant evolution [12,13,14], and at least 47% of flowering plants have experienced a polyploidy event throughout their evolutionary history [15]. WGD events may have played a very important role in the generation of plant diversity in two different ways: directly, due to speciation connected to polyploidization [15], and indirectly, as polyploid lineages often have higher diversification rates [16,17]. Polyploidy might have significant effects on the biochemistry, ecophysiology and morphology of plants [18,19]. These phenotypic changes may be due to altered gene expression, increased variation in dosage-regulated gene expression, modified regulatory interaction, epigenetic changes, gene redundancy or heterozygosity [20,21]. Taking this into account, and with the objective of studying these biological processes, additional research focusing on finding stable reference genes between different ploidy levels to perform gene expression analysis is required [22,23].

*Dianthus* is a Eurasian genus belonging to Caryophyllaceae, which has a striking diversity with more than 300 species that have been recently originated [24]. This genus is known for its outstanding horticultural relevance as several species (pinks and carnations) have been cultivated for centuries and they were common in Ancient Greek and Roman times. Polyploidy is widespread in this genus, with 67% of diploid species, 18.7% tetraploid, 6.6% hexaploid and 7.7% corresponding to different cytotypes [25]. *Dianthus broteri* represents the largest polyploid series known in the genus with four different cytotypes (2×, 4×, 6× and 12×) [19]. This endemic complex to the Iberian Peninsula extends along from eastern Spain to southern Portugal. *Dianthus broteri* appears to be a great study system to investigate the importance of polyploidy in evolution [26]. Cytotypes do not coexist and occupy disjunct geographic ranges with markedly different environmental conditions [27]. Furthermore, cytotypes show divergence in morphological traits, vegetative and reproductive organs [28], and photochemical responses [29]. The inter-cytotype differentiation pattern in this complex also encompasses global methylation which could affect the global regulation of gene expression [30]. Therefore, *Dianthus broteri* is a suitable model study system to evaluate the impact of polyploidy in gene expression.

The aim of this study is to determine suitable reference genes in *Dianthus broteri* to compare gene expression among ploidy levels and different plant tissues. We selected thirteen candidate reference genes based on preceding literature reports about genes with constitutive expression in a wide range of plant species. These candidates were evaluated for their inherent role as internal normalization controls in leaf and petal tissues for the four different cytotypes of *Dianthus broteri* (2×, 4×, 6× and 12×).

## 2. Results

### 2.1. Verification of Primer Specificity and Efficiency of Candidate Reference Genes

A total of 13 candidate reference genes were subjected to qPCR to verify their specificity and efficiency: EF1α, TIP41, UBQ10, UBQ3, SAMDC, PP2A, TIF5A (also can be found as eIF-5A in bibliography), H3, PR13S, GAPDH, ACT7, TUA and TUB (Table 1). After 55 cycles of amplification, qPCR yielded the melting curve which showed a single peak from all samples. The specificity of amplicons and the absence of dimers were validated (Supplementary Materials Figure S1). All fourteen primer pairs had similar efficiency (E) values, ranging between 1.760 and 2.187 (Table 1).

**Figure 1:**
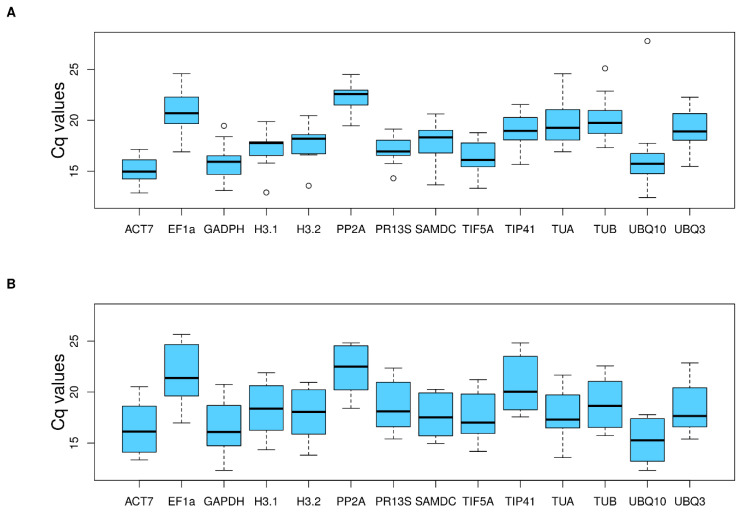
Expression profile of candidate reference genes. Values are provided as quantification cycle (Cq) in leaves (**A**) and petals (**B**). The median, 25th–75th percentiles and maximum-minimum Cq value for each gene are represented. Amplification curves of the candidate genes in leaves and petals are available in Supplementary Materials Figures S1 and S2.

The expression profiles of the 13 candidate reference genes were represented using the quantification cycle (Cq) value. This parameter is defined as the number of cycles needed for the fluorescent signal transcending the background fluorescence [8]. Therefore, a low Cq value corresponds to a high expression level. Although initially, the added amounts of cDNA in the qPCR reaction were the same, the candidate reference genes displayed a relatively wide range of Cq values which varied across different levels of ploidy in each tissue. In the leaf tissue, the candidate reference genes showed a high variation in expression levels, ranging from a minimum Cq value of 15.145 for ACT7 to 22.33 for PP2A (Figure 1A, Supplementary Materials Figure S2). In petals, the Cq value ranged from 15.31 for UBQ10 to 22.19 for PP2A (Figure 1B, Supplementary Materials Figure S3).

### 2.2. Determination of Expression Stability of Candidate Reference Genes in Leaf Tissue

**Figure 2:**
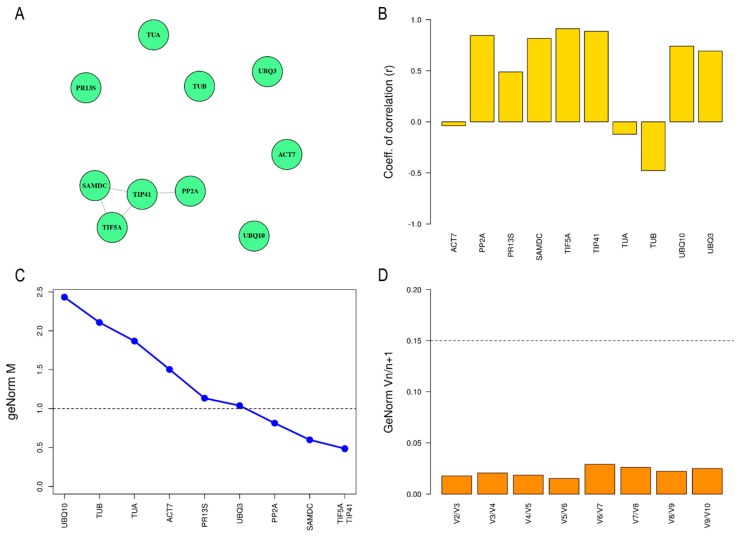
Analysis of reference genes stability in leaf tissue based on different algorithms. (**A**) Equivalence graph according to SARP.compo package: each node represents a candidate gene and nodes connected implies that there is an equivalence between them. (**B**) Representation of correlation coefficients (r value) of the 10 candidate reference genes in leaf tissue calculated by BestKeeper software. (**C**) Average expression of stability (M value) of the ten candidate reference genes provided by geNorm approach. Dotted line indicates cut-off value of one below which a reference gene is considered stable enough to be used in normalization. (**D**) Determination of the optimal number of reference genes required for normalization in *Dianthus broteri* leaf tissue. The cut-off value of 0.15 is represented by the dotted line.

Firstly, the 13 candidate reference genes were tested on the different cytotypes (2×, 4×, 6× and 12×) in leaf tissue. EF1a, GAPDH, H3.1 and H3.2 showed differential expression among ploidy levels (*p*-value < 0.01) in the Bayesian analysis with MCMC.qpcr (Supplementary Materials Table S1). Therefore, the latter genes were discarded as reference genes to study gene expression in *D. broteri*. In contrast, the Bayesian analysis determined that ACT7, PP2A, PR13S, SAMDC, TIF5A, TIP41, TUA, TUB UBQ10 and UBQ3 remained stable at expression among all ploidy levels. These 10 stable genes were subjected to equivalence tests using the SARP.compo R package, with a p-value cut-off of 0.25 (based in simulations) for the node connection [33]. For leaf tissue, the set of genes formed by PP2A, TIP41, TIF5a and SAMDC were equivalent in expression as shown in Figure 2A.

In addition, the stability in expression of the remaining candidate reference genes after MCMC analysis was determined with BestKeeper and geNorm. ACT7, TUA and TUB had a negative BestKeeper r index indicating that they were not suitable as reference genes (Figure 2B and Table 2). The remaining candidate genes had a positive r index. TIP41, TIF5a and PP2A genes (with r value close to 0.9) stand out as the best reference genes based on this algorithm. In the geNorm analysis, candidate reference genes were ranked by calculating stability value (M) where the magnitude of M value has a negative correlation with gene stability. In this work, the M values of the 10 candidate reference genes in leaf tissue ranged from 0.47 in TIF5A and TIP41 to 2.64 in TUA (Figure 2C and Table 2), Therefore, only TIP41, TIF5a, SAMDC and PP2A showed M values lower than one and they could be considered as stable candidate reference genes.

### 2.3. Determination of Expression Stability of Candidate Reference Genes in Petal Tissue

**Figure 3:**
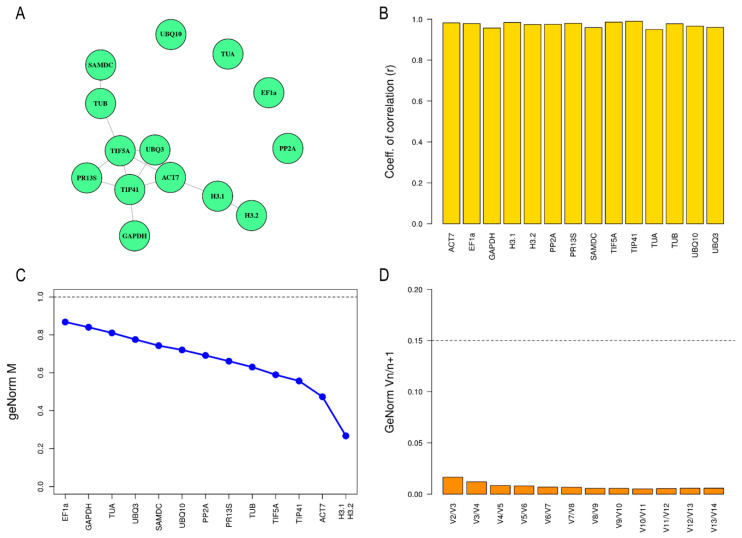
Analysis of reference genes stability in petal tissue based on different algorithms. (**A**) Equivalence graph obtained from 13 candidate reference genes in the petal tissue, where the node connected implies that there is an equivalence between these candidate reference genes, according to SARP.compo. (**B**) Representation of correlation coefficients (r value) of the ten candidate reference genes in petal tissue calculated by BestKeeper software. (**C**) Average expression of stability (M value) of thirteen candidate reference genes provided by geNorm approach. Dotted line indicates cut-off value of one below which a reference gene is considered stable enough to be used in normalization. (**D**) Determination of the optimal number of reference genes required for normalization in *Dianthus broteri* petal tissue. Dotted lined determine cut-off of 0.15 above which another reference gene will need to be introduced for appropriate normalization.

The same workflow to analyze candidate reference genes for leaf tissue was used for petal tissue. Firstly, no candidate genes showed significant differences in expression among ploidy levels in the Bayesian analysis using the MCMC.qpcr (Supplementary Materials Table S2). Therefore, all candidate genes were appropriate as reference genes. Subsequently, the 13 candidate reference genes were subjected to a network analysis using equivalence tests using the same p-value of 0.25 as in leaf tissue. The network graph (Figure 3A) suggested that H3.1, H3.2, ACT7, UBQ3, TIP41, TIF5A, GAPDH, TUB, SAMDC and PR13S showed an equivalent expression. Specifically, TIP41, TIF5a, UBQ3 and ACT7 were the most equivalent in expression pattern.

Furthermore, the expression stability in petals was also studied with BestKeeper and geNorm. In BestKeeper, the 13 candidate reference genes had an r-value very close to one (Figure 3B and Table 3) making them suitable as reference genes. The geNorm approach provided us with M values that ranged from 0.27 in H3.1 and H3.2 to 0.86 in EF1a and the most stable candidate genes for this index were H3.1, H3.2, ACT7 and TIP41 (Figure 3C and Table 3). Although according to BestKeeper and geNorm methods all the initial 13 candidate genes were stable enough to be considered as appropriate reference genes for normalization.

### 2.4. Determination of Optimal Number of Reference Genes

The geNorm software was also used to compare paired differences between candidate references genes to evaluate the optimal number of required internal reference genes. When the pairwise variation (Vn/n+1) is lower than 0.15, another internal reference gene for correction is not needed, and the optimal number of internal reference genes were two [34,35]. In both leaf and petal tissue, the Vn/Vn+1 value was lower than 0.15 (Figure 2D and Figure 3D). Consequently, adding an extra reference is not necessary since it does not make a significant contribution to normalization. Therefore, the recommended number of reference genes would be two.

## 3. Discussion

### 3.1. Reference Gene Stability among Tissues and Ploidy Levels

Gene expression analysis is one of the most effective strategies for understanding many biological processes and, currently, qPCR is an accurate tool to study the changes in gene expression when normalized using correct reference genes [36]. Assuming that an absolutely stable reference gene (under all experimental conditions, tissues and species) does not exist, an ideal reference gene should have a stable expression in a wide range of environmental conditions, tissues and growth stages. At the present time, only four studies have investigated reference genes for allopolyploid species [10,11,37,38], and only one has focused on autopolyploid species [23].

Here, we assessed the potential of 13 candidate genes to be considered as reference gene for qPCR normalization in *Dianthus broteri* autopolyploid complex. We tested their expression and stability across two tissues (leaf and petal) and four ploidy levels. The statistical analyses of geNorm and BestKeeper provided similar (but not equal) reference genes rankings for the leaf and petal tissues. The most stable candidate reference genes in the leaf were TIF5A, TIP41, PP2A and SAMDC. Meanwhile, in the petal tissue, H3.1, ACT7, TIP41, TIF5A were identified as the most stable candidate genes. Therefore, we suggest that the best reference genes to compare the four different ploidy levels for both tissues in *D. broteri* are TIP41 and TIF5A.

TIP41 has been proved to be useful both as general reference gene and among different tissues in other plant species such as *Cucumis sativus* (cucumber), *Lycoris aurea* and *Phyllostachys edulis* [39,40,41]. TIF5A has also been used as reference gene in *Zanthoxylum bungeanum Maxim* (Chinese prickly) [42], and *Populus bejingensis* (poplar) to study the Poplar/Canker disease interaction system [43]. Most importantly, a recent study evaluated reference genes in *Dianthus caryophyllus* [9], in which TIP41 stood out as an appropriate stable reference gene in general and between different tissues, while TIF5A, together with EF1α, was the most appropriate one to study gene expression under stress conditions (hormone treatment, heavy metal, salt, heat, cold, flooding and drought) [9]. Although most cultivars of *D. caryophyllus* are diploids, a few are tetraploid and triploids [44], the effect of polyploidy *on D. caryophyllus* gene expression has not been studied. Therefore, we propose TIP41 and TIF5A as candidate reference genes to study gene expression among ploidy levels in *D. caryophyllus* leaf and petal tissues. Other genes (like H3.1, ACT7, PP2A and SAMDC) could also be suitable depending on the tissue or experimental conditions. Nevertheless, the conventional reference genes UBQ10 and GAPDH were discarded as reference genes in leaves because they showed differential expression among cytotypes of *Dianthus broteri*. For example, GAPDH (involved in the production of energy during photosynthesis) showed differential expression in concordance with previous studies showing differences in photochemichal responses [29].

### 3.2. Gene Expression Attenuation Enable the Comparison among the Four Ploidy Levels

In this study, special emphasis has been placed on the selected reference gene maintaining a stable profile expression at different ploidy levels. Polyploidy events may influence the regulation of gene expression due to epigenetic control, genomic rearrangement or dosage effects [23]. Several studies suggest that the stability of the reference genes is influenced by the ploidy level, especially dosage effects or compensations are triggered by genome duplication [23]. Although duplication by polyploidy increases the dosage of all genes and so should not affect the balance, for every gene duplicated by polyploidy, a diversity of transcriptional dosage responses (changes in expression associated with changes in gene dosage) is feasible [45]. Some dosage-dependent genes will increase (or decrease) the absolute gene expression according to the duplication. Meanwhile, gene expression can be maintained stable irrespectively of the ploidy level (gene copy) by dosage compensation, considering that the excess of gene expression, and consequent protein synthesis, can be detrimental due to the waste of energy and resources [46]. This waste would be evident in housekeeping genes which are constitutively expressed. Furthermore, the gene dosage balance hypothesis (DBH) determines that there is a selection that acts against the duplication of genes of the central macromolecular complexes and in signaling/transcriptional networks due to stoichiometric imbalance. This imbalance is established when the loss of copies of many genes takes place with respect to the rest of the genes that remain duplicates in the diploidization process [47,48]. Gene expression attenuation can be mediated by epigenetics changes after polyploidization [20]. Accordingly, an increased amount and variation in epigenetic marks have been shown in *Dianthus broteri* [49]. In addition, a moderate genomic diploidization was evident in the different cytotypes of *Dianthus broteri* as regards the loss of monoploid genome size [26,50]. The phenotypic diploidization found in several functional traits also suggests that purifying selection is acting in this polyploid complex [50]. Therefore, the dosage compensation of the selected reference genes could have also been driven by selection to maintain the genomic balance.

Supporting the DBH hypothesis, the selected reference genes encode macromolecular complexes, including transcription factors or signal transduction pathways. For example, TIF5A encodes a highly conserved initiation transcription factor regulating cell division, cell growth, and cell death [51], and TIP41 is an ABA-responsive gene encoding a regulatory protein of PP2A, a complex involved in the regulation of growth and development [52]. Likewise, other candidate genes which stood out as stable reference genes also have this key role in metabolism: SAMDC is involved in the polyamine biosynthetic pathway, H3.1 is implicated in the structural conformation of the chromosome and ACT7 that results in a filamentous protein involved in the cytoskeleton structure [52].

## 4. Materials and Methods

### 4.1. Plant Material and Growth Conditions

For each one of the four ploidy levels of *Dianthus broteri* (2×, 4×, 6× and 12×), three individuals belonging to three different populations were selected as biological replicates, with a total of 12 samples for each tissue (leaves and petals). The selected individuals for the experiment were kept in a climate chamber from the greenhouse services of the research center “Centro de Investigación, Tecnología e Innovación de la Universidad de Sevilla” (CITIUS II), in which the controlled environmental conditions were optimal to grow up and bloom (16 h light 25 °C/8 h dark 21 °C regimen with 50% humidity).

### 4.2. RNA Extraction and cDNA Synthesis

For the RNA extraction of each individual, a 100 mg sample from each tissue (leaf or petal) was used. The sample was immediately frozen in liquid nitrogen and then disrupted using a bead miller (TissueLyser II). RNA was extracted and isolated using the commercial kit Direct-zol RNA Miniprep (Zymo Research), and then quantified by Qubit fluorometric quantification and Nanodrop spectrophotometer, to check the quantity and the quality of the RNA obtained. Resulting RNA was reverse transcribed using the iScript cDNA synthesis kit (Bio-Rad, Hercules, CA, USA). In total 1 µg of RNA was reverse transcribed according to the protocol, and then quantified by Qubit fluorometric quantification.

### 4.3. Identification of Candidate Reference Genes and Efficiency Evaluation

Reference genes were chosen based upon prior studies of reference genes on different plant species, with a total of 13 genes selected as candidate reference genes for analysis of expression stability (Table 1). In the case of the H3 gene, we tested two different sets of primers, H3.1 and H3.2. Primers pairs sequences were taken from literature and tested their homology to *Dianthus* using the BLAST tool from the *Dianthus caryophyllus* genome database, Carnation DB [53]. Sequences were modified when needed to increase specificity, and melting temperature, primer dimers and hairpin were verified.

Primer efficiency is a parameter that is mainly evidence of how well the qPCR reaction has performed [54]. The efficiency of each candidate reference gene was examined through standard curve analysis. Each primer pair efficiency (E) was calculated performing a quantitative real-time PCR (qPCR) analysis carried out on a LightCycler^®^ 480 Instrument II (Roche Molecular System, Germany). A cDNA sample from a 2x individual (diploid) was used for the efficiency experiment. The sample was diluted in a serial gradient adding molecular water (1:0, 1:2, 1:4, 1:6, 1:8 and 1:10), with a starting concentration of 12 ng/μL. For each qPCR reaction 4 μL of the sample, 5 μL of the iTaq Universal SYBR Green Supermix (Bio-Rad, Hercules, CA, USA), and 0.5 μL of each primer (forward and reverse) was added up to a final volume of 10 μL. For each primer pair, four technical replicates were performed. The qPCR program run consisted of an initial denaturation step at 95 °C for 5 min followed by amplification and quantification cycles repeated 55 times at 95 °C for 10 s, 60 °C for 10 s and 72 °C for 15 s [7]. After 55 amplification cycles, the LightCycler System DNA melting curve analysis was performed. In this program, the temperature increased 65–97 °C with a heating rate of 0.11 °C/s and continuous fluorescence measurement.

### 4.4. Determination of Expression Stability of Reference Genes in Reproductive and Vegetative Tissues by Statistical Analyses

The expression of the 13 selected reference genes was analyzed by qPCR in three different biological samples of each ploidy level (12 samples in total) in two different tissues, leaf and petal. For each primer pair, three cDNA technical replicates of the 12 biological samples were used in the qPCR reaction with a concentration of 12 ng/μL. qPCR ran under the same conditions used to test the efficiency mentioned above [7]. The expression stability in different ploidy levels of *D. broteri* was evaluated using different approaches. All the statistical analysis was performed using R statistical software vers. 3.6.0 [55]. Firstly, differences in gene expression among ploidy levels for all the candidate reference genes were assessed using MCMC.qpcr R package with default parameters [56]. This software estimate gene expression of each gene based on the efficiency and Cq values for the entire data set, including the random effects common to all the genes [57]. The expression of the genes that were stable between ploidy levels was then tested using equivalence tests and a network analysis, implemented in the SARP.compo R package vers. 0.1.5 [58]. To select the candidate genes that are stable enough to be used as reference genes, this method provides a network graph based on all pairwise ratio comparisons which represents the set of genes that maintain a common and equivalent expression between them [33]. The *p*-value matrix to construct the graph was created following SARP.compo package documentation, with a Delta value of 1 and a *p*-value cut-off of 0.25 [33,58].

Finally, to validate the expression stability of candidate reference genes two statistical algorithms were used, geNorm and BestKeeper, implemented in the ctrlGene R package [59], which was used with default parameters for both analyses. GeNorm is an algorithm that carries out two analyses: (1) determining the most stable reference gene based on the geometric mean of reference genes; (2) informing whether more reference genes are required [60]. GeNorm provides an “M” value (stability measure) which represents the variation of a certain gene versus all other normalizing genes. The M value is defined as the average pairwise variation between a gene and all other reference genes. Recent evidence suggests that if the “M” value is less than one indicates high stability expression so it will be an appropriate reference gene. This same statistical method was also used to analyze the variation of pairs of candidate reference genes with the purpose of determining the optimal number of reference genes for an accurate subsequence normalization. Generally, it is recommended to use at least two reference genes, although more could be used if the “PV” or “(Vn/n+1)” value (paired variation) exceeds the threshold PV of 0.15 [61]. Additionally, BestKeeper [62] was used to select the best reference genes based on expression stability, taking into account three statistical variables: standard deviation (SD), Pearson coefficient of correlation (r) and percentage covariance (CV) [63]. BestKeeper determines an index which is calculated from the Cq arithmetic mean of each candidate reference gene, being the most stable reference genes those having a higher r-value [64,65].

## 5. Conclusions

Our results reinforce the need of searching for suitable reference genes taking into consideration polyploidy as a cause of differential gene expression. We evaluated the stability of 13 candidate reference genes with the aim of normalizing the gene expression in the leaf and petal tissues of *Dianthus broteri* polyploid complex. Overall, the results obtained showed us the most stable candidate genes were those having an important role in plant metabolism. The reference genes identified in this research are useful tools to investigate polyploid evolution in *Dianthus broteri*. In addition, provided that the selected reference genes in *D. broteri* can be extrapolated to other polyploid plant species, particularly among polyploid cultivars of ornamental *Dianthus*, this work could help to study gene expression differences in polyploids.

## Figures and Tables

**Table 1:** Reference genes primer sequences and amplicon characteristics in *Dianthus broteri*. H3.1 and H3.2 are different primers of the same gene.

Gene	Gene Name	Accession No. (Carnation DB)	Primer Sequence	Efficiency	Product Size (bp)	Tm (°C)	References
EF1α	Elongation factor 1α	Dca5900.1	F: ACCCCGACAAGATCCCATTT R: TGGTCAAGGGCCTCAAGTAG	2.129	115	56.94 56.99	[9]
TIP41	Phosphatase activator	Dca43498.1	F: GACACTCGTATGCATTGCGT R: CTCGAACTGATGACGCTTGG	2.187	152	57.07 57.08	[9]
UBQ10	Ubiquitin 10	Dca41829.1	F: CCATTTGGTGTTGCGTCTCA R: TCGCTGCTCTCCACTTCC	1.928	90	57.08 56.44	[9]
UBQ 3	Ubiquitin 3	Dca119.1	F: GCGTATGAGCAACGAGTCAG R: AGGATCTGCTTTACCCACCA	1.953	150	57.17 56.27	[5]
SAMDC	Adenosylmethionine decarboxylase gene	Dca28802.1	F: AAACCAACTACGACGACCCT R: CCGATGCCTTCTCCTTGTCA	2.021	72	56.95 57.75	[9]
PP2A	Protein phosphatase 2A	Dca33231.1	F: TCGAGCAGTTGATGGAGTGT R:ACTCTTCAACCAAAACCGCC	1.992	87	59.03 58.97	[9]
TIF5A	Translation initiation factor	Dca33327	F: GGCGGGGAAAGACTTGATTC R: CTACTTGCCACCACTAACGT	1.935	93	58.90 58.94	[9]
H3.1	Histone 3	Dca5219.1	F: GGAGGAGTGAAGAAGCCACA R: GTGCCAACACAGCATGACTC	1.868	178	57.3 57.81	[5]
H3.2	Histone 3	Dca5219.1	F: CACAGGTACCGTCCTGGAAC R: GTGCCAACACAGCATGACTC	1.882	160	58.06 57.81	[5]
PR13S	Ribosomal protein	Dca22015.1	F: AATCCCCGAGGACTTGTACC R:ACAAGAGTACTGGCGGTGGT	2.051	201	56.77 59.09	[5]
GAPDH	Glyceraldehyde 3 phosphate dehydrogenase	Dca8698.1	F: GGCCAAGGTTATCAATGACAG R: CCTTCCACCTCTCCAGTCCT	2.008	120	54.18 57.12	[7]
ACT7	Actin	Dca37612.1	F: CGGTGGCTCTATCCTCGCTT R: TTCCTGTGGACGATTGACGG	1.854	94	58.7 57.02	[31]
TUA	Tubulin α	Dca60406.1	F: ACATGGCTTGCTGTCTGATG R: TGGGGGCTGGTAGTTGATAC	2.042	142	55.51 55.69	[7]
TUB	Tubulin β	Dca39629.1	F: TGTTGCATCCTGGTACTGCT R: GGCTTTCTTGCACTGGTACAC	1.760	73	56.22 57.00	[32]

**Table 2:** Gene expression stability in leaf tissue ranked by BestKeeper and geNorm algorithms.

	BestKeeper		geNorm	
Ranking	Gene	r Value	Gene	M Value
1	TIF5A	0.893	TIF5A	0.471
2	TIP41	0.868	TIP41	0.471
3	PP2A	0.851	SAMDC	0.701
4	SAMDC	0.776	PP2A	0.845
5	UBQ10	0.728	UBQ3	1.042
6	UBQ3	0.676	PR13S	1.137
7	PR13S	0.441	ACT7	1.512
8	ACT7	−0.015	TUB	1.933
9	TUA	−0.268	UBQ10	2.308
10	TUB	−0.431	TUA	2.648

**Table 3:** Ranking of stability for the candidate reference genes in petal tissue according to BestKeeper and geNorm.

	BestKeeper		geNorm	
Ranking	Gene	r Value	Gene	M Value
1	TIP41	0.990	H3.1	0.267
2	TIF5A	0.986	H3.2	0.267
3	H3.1	0.984	ACT7	0.473
4	ACT7	0.982	TIP41	0.557
5	PR13S	0.980	TIF5A	0.589
6	EF1a	0.979	TUB	0.630
7	TUB	0.978	PR13S	0.661
8	PP2A	0.975	PP2A	0.691
9	H3.2	0.974	UBQ10	0.720
10	UBQ10	0.966	SAMDC	0.743
11	UBQ3	0.960	UBQ3	0.776
12	SAMDC	0.959	TUA	0.810
13	GAPDH	0.957	GAPDH	0.840
14	TUA	0.950	EF1a	0.868

## Data Availability

Not applicable.

## Supplementary Materials

The following supporting information can be downloaded at: https://www.mdpi.com/article/10.3390/plants11040518/s1, Figure S1: Melting curves for thirteen candidate reference genes in *Dianthus broteri*; Figure S2: qPCR amplification curves for thirteen candidate reference genes in *Dianthus broteri* leaves; Figure S3: qPCR amplification curves for thirteen candidate reference genes in *Dianthus broteri* petals; Table S1: Results of the Bayesian analysis for pairwise differential expression among cytotypes (MCMC.qpcr package) in the leaf tissue; Table S2: Results of the Bayesian analysis for pairwise differential expression among cytotypes (MCMC.qpcr package) in the petal tissue.
