# Supplementary material for: Evaluation of Reference Genes in the Polyploid Complex Dianthus broteri (Caryophyllaceae) Using qPCR
Source: Plants (Basel). 2022 Feb 14;11(4):518. doi: 10.3390/plants11040518 (PMC8878694; doi:10.3390/plants11040518)
Supplement: Supplementary file 1 [file plants-11-00518-s001.zip › supplementary_tables_figslegends.pdf]

**Supplementary Table S1:** Results of the Bayesian analysis for pairwise differential expression among cytotypes (MCMC.qpcr package) in the leaf tissue. The *p* values of each candidate reference gene and pairwise comparison are shown.

**ACT7**

| pvalue \ difference | 2X | 4X | 6X | 12X |
|---|---|---|---|---|
| 2X | NA | 0.0859 | 1.373 | 0.2 |
| 4X | 0.939 | NA | 1.287 | 0.1 |
| 6X | 0.207 | 0.2623 | NA | -1.2 |
| 12X | 0.847 | 0.9119 | 0.31 | NA |

**EF1a**

| pvalue \ difference | 2X | 4X | 6X | 12X |
|---|---|---|---|---|
| 2X | NA | -0.228 | -2.03 | -5.1 |
| 4X | 0.876 | NA | -1.8 | -4.9 |
| 6X | 0.162 | 0.2362 | NA | -3.1 |
| 12X | 6E-04 | 0.0012 | 0.042 | NA |

**GAPDH**

| pvalue \ difference | 2X | 4X | 6X | 12X |
|---|---|---|---|---|
| 2X | NA | 2.1637 | 3.161 | 3.5 |
| 4X | 0.063 | NA | 0.997 | 1.3 |
| 6X | 0.004 | 0.4001 | NA | 0.3 |
| 12X | 0.002 | 0.2536 | 0.764 | NA |

**H3.1**

| pvalue \ difference | 2X | 4X | 6X | 12X |
|---|---|---|---|---|
| 2X | NA | 1.6081 | -1.13 | -1.4 |
| 4X | 0.121 | NA | -2.74 | -3 |
| 6X | 0.258 | 0.0096 | NA | -0.3 |
| 12X | 0.174 | 0.0069 | 0.811 | NA |

**H3.2**

| pvalue \ difference | 2X | 4X | 6X | 12X |
|---|---|---|---|---|
| 2X | NA | 5.4235 | 2.391 | 2.6 |
| 4X | 0.005 | NA | -3.03 | -2.8 |
| 6X | 0.203 | 0.1132 | NA | 0.2 |
| 12X | 0.173 | 0.1395 | 0.916 | NA |

**PP2A**

| pvalue \ difference | 2X | 4X | 6X | 12X |
|---|---|---|---|---|
| 2X | NA | -0.028 | -1.34 | -1.1 |
| 4X | 0.978 | NA | -1.31 | -1 |
| 6X | 0.177 | 0.1979 | NA | 0.3 |
| 12X | 0.311 | 0.3475 | 0.792 | NA |

**PR13S**

| pvalue \ difference | 2X | 4X | 6X | 12X |
|---|---|---|---|---|
| 2X | NA | 1.1517 | -1.45 | -1.1 |
| 4X | 0.266 | NA | -2.61 | -2.2 |
| 6X | 0.151 | 0.0126 | NA | 0.4 |
| 12X | 0.308 | 0.039 | 0.721 | NA |

**SAMDC**

| pvalue \ difference | 2X | 4X | 6X | 12X |
|---|---|---|---|---|
| 2X | NA | 0.66 | -1.5 | -1.212 |
| 4X | 0.558 | NA | -2.1 | -1.869 |
| 6X | 0.186 | 0.06 | NA | 0.265 |
| 12X | 0.278 | 0.11 | 0.81 | NA |

**TIF5A**

| pvalue \ difference | 2X | 4X | 6X | 12X |
|---|---|---|---|---|
| 2X | NA | 0.04 | -1.9 | -1.311 |
| 4X | 0.971 | NA | -1.9 | -1.35 |
| 6X | 0.069 | 0.07 | NA | 0.558 |
| 12X | 0.193 | 0.21 | 0.6 | NA |

**TIP41**

| pvalue \ difference | 2X | 4X | 6X | 12X |
|---|---|---|---|---|
| 2X | NA | 0.11 | -1.8 | -1.577 |
| 4X | 0.915 | NA | -1.9 | -1.691 |
| 6X | 0.081 | 0.08 | NA | 0.251 |
| 12X | 0.137 | 0.12 | 0.82 | NA |

**TUA**

| pvalue \ difference | 2X | 4X | 6X | 12X |
|---|---|---|---|---|
| 2X | NA | -1.23 | 1.99 | 0.557 |
| 4X | 0.488 | NA | 3.22 | 1.787 |
| 6X | 0.261 | 0.08 | NA | -1.437 |
| 12X | 0.766 | 0.35 | 0.44 | NA |

**TUB**

| pvalue \ difference | 2X | 4X | 6X | 12X |
|---|---|---|---|---|
| 2X | NA | 0.4 | 2.5 | 1.967 |
| 4X | 0.753 | NA | 2.1 | 1.565 |
| 6X | 0.038 | 0.11 | NA | -0.536 |
| 12X | 0.127 | 0.24 | 0.68 | NA |

**UBQ10**

| pvalue \ difference | 2X | 4X | 6X | 12X |
|---|---|---|---|---|
| 2X | NA | -3.32 | -2.2 | -1.12 |
| 4X | 0.029 | NA | 1.1 | 2.201 |
| 6X | 0.141 | 0.46 | NA | 1.098 |
| 12X | 0.481 | 0.15 | 0.48 | NA |

**UBQ3**

| pvalue \ difference | 2X | 4X | 6X | 12X |
|---|---|---|---|---|
| 2X | NA | 0.84 | -2.3 | -1.764 |
| 4X | 0.409 | NA | -3.1 | -2.604 |
| 6X | 0.026 | 0.022 | NA | 0.489 |
| 12X | 0.084 | 0.02 | 0.64 | NA |

**Supplementary Table S2:** Results of the Bayesian analysis for pairwise differential expression among cytotypes (MCMC.qpcr package) in the petal tissue. The *p* values of each candidate reference gene and pairwise comparison are shown.

| | | difference | | | |
|---|---|---|---|---|---|
| | pvalue | 2X | 4X | 6X | 12X |
| ACT7 | 2X | NA | 0.734 | -1.79 | -0.22 |
| | 4X | 0.742 | NA | -2.52 | -0.95 |
| | 6X | 0.439 | 0.265 | NA | 1.573 |
| | 12X | 0.923 | 0.669 | 0.476 | NA |

| | | difference | | | |
|---|---|---|---|---|---|
| | pvalue | 2X | 4X | 6X | 12X |
| EF1a | 2X | NA | -1.23 | -3.95 | -3.15 |
| | 4X | 0.584 | NA | -2.72 | -1.92 |
| | 6X | 0.088 | 0.23 | NA | 0.799 |
| | 12X | 0.158 | 0.389 | 0.718 | NA |

| | | difference | | | |
|---|---|---|---|---|---|
| | pvalue | 2X | 4X | 6X | 12X |
| GAPDH | 2X | NA | 0.574 | -2.64 | -1.46 |
| | 4X | 0.799 | NA | -3.22 | -2.03 |
| | 6X | 0.257 | 0.16 | NA | 1.186 |
| | 12X | 0.515 | 0.368 | 0.596 | NA |

| | | difference | | | |
|---|---|---|---|---|---|
| | pvalue | 2X | 4X | 6X | 12X |
| H3.1 | 2X | NA | -0.06 | -2.23 | -0.43 |
| | 4X | 0.979 | NA | -2.18 | -0.37 |
| | 6X | 0.334 | 0.334 | NA | 1.805 |
| | 12X | 0.845 | 0.864 | 0.414 | NA |

| | | difference | | | |
|---|---|---|---|---|---|
| | pvalue | 2X | 4X | 6X | 12X |
| H3.2 | 2X | NA | -0.47 | -2.48 | -0.85 |
| | 4X | 0.833 | NA | -2.01 | -0.38 |
| | 6X | 0.283 | 0.369 | NA | 1.631 |
| | 12X | 0.7 | 0.863 | 0.457 | NA |

| | | difference | | | |
|---|---|---|---|---|---|
| | pvalue | 2X | 4X | 6X | 12X |
| PP2A | 2X | NA | -0.63 | -2.76 | -1.96 |
| | 4X | 0.774 | NA | -2.13 | -1.32 |
| | 6X | 0.233 | 0.35 | NA | 0.806 |
| | 12X | 0.371 | 0.551 | 0.716 | NA |

| | | difference | | | |
|---|---|---|---|---|---|
| | pvalue | 2X | 4X | 6X | 12X |
| PR13S | 2X | NA | 0.113 | -3.48 | -1.79 |
| | 4X | 0.959 | NA | -3.59 | -1.91 |
| | 6X | 0.131 | 0.109 | NA | 1.684 |
| | 12X | 0.417 | 0.386 | 0.441 | NA |

| | | difference | | | |
|---|---|---|---|---|---|
| | pvalue | 2X | 4X | 6X | 12X |
| SAMDC | 2X | NA | 0.4899 | -2.33 | -1.7 |
| | 4X | 0.823 | NA | -2.82 | -2.2 |
| | 6X | 0.313 | 0.2144 | NA | 0.6 |
| | 12X | 0.427 | 0.3164 | 0.79 | NA |

| | | difference | | | |
|---|---|---|---|---|---|
| | pvalue | 2X | 4X | 6X | 12X |
| TIF5A | 2X | NA | 0.6389 | -2.11 | -0.8 |
| | 4X | 0.772 | NA | -2.74 | -1.4 |
| | 6X | 0.363 | 0.2242 | NA | 1.33 |
| | 12X | 0.724 | 0.5188 | 0.54 | NA |

| | | difference | | | |
|---|---|---|---|---|---|
| | pvalue | 2X | 4X | 6X | 12X |
| TIP41 | 2X | NA | 0.5115 | -3.51 | -1.3 |
| | 4X | 0.818 | NA | -4.02 | -1.9 |
| | 6X | 0.138 | 0.0751 | NA | 2.17 |
| | 12X | 0.543 | 0.4018 | 0.33 | NA |

| | | difference | | | |
|---|---|---|---|---|---|
| | pvalue | 2X | 4X | 6X | 12X |
| TUA | 2X | NA | 0.2039 | -2.69 | -1.4 |
| | 4X | 0.927 | NA | -2.89 | -1.6 |
| | 6X | 0.249 | 0.2103 | NA | 1.25 |
| | 12X | 0.512 | 0.4609 | 0.58 | NA |

| | | difference | | | |
|---|---|---|---|---|---|
| | pvalue | 2X | 4X | 6X | 12X |
| TUB | 2X | NA | -0.026 | -2.27 | -1.5 |
| | 4X | 0.991 | NA | -2.24 | -1.5 |
| | 6X | 0.326 | 0.3229 | NA | 0.74 |
| | 12X | 0.485 | 0.4978 | 0.74 | NA |

| | | difference | | | |
|---|---|---|---|---|---|
| | pvalue | 2X | 4X | 6X | 12X |
| UBQ10 | 2X | NA | -0.49 | -2.04 | -1.7 |
| | 4X | 0.824 | NA | -1.55 | -1.2 |
| | 6X | 0.381 | 0.495 | NA | 0.38 |
| | 12X | 0.451 | 0.5961 | 0.86 | NA |

| | | difference | | | |
|---|---|---|---|---|---|
| | pvalue | 2X | 4X | 6X | 12X |
| UBQ3 | 2X | NA | 0.956 | -2.06 | -0.7 |
| | 4X | 0.667 | NA | -3.01 | -1.6 |
| | 6X | 0.379 | 0.1846 | NA | 1.39 |
| | 12X | 0.759 | 0.4645 | 0.53 | NA |

**Supplementary Figure S1:** Melting curves for thirteen candidate reference genes in *Dianthus broteri*.

**Supplementary Figure S2:** qPCR amplification curves for thirteen candidate reference genes in *Dianthus broteri* leaves.

**Supplementary Figure S3:** qPCR amplification curves for thirteen candidate reference genes in *Dianthus broteri* petals.
