# Supplementary figures and images for: Evaluation of Reference Genes in the Polyploid Complex Dianthus broteri (Caryophyllaceae) Using qPCR
Source: Plants (Basel). 2022 Feb 14;11(4):518. doi: 10.3390/plants11040518 (PMC8878694; doi:10.3390/plants11040518)

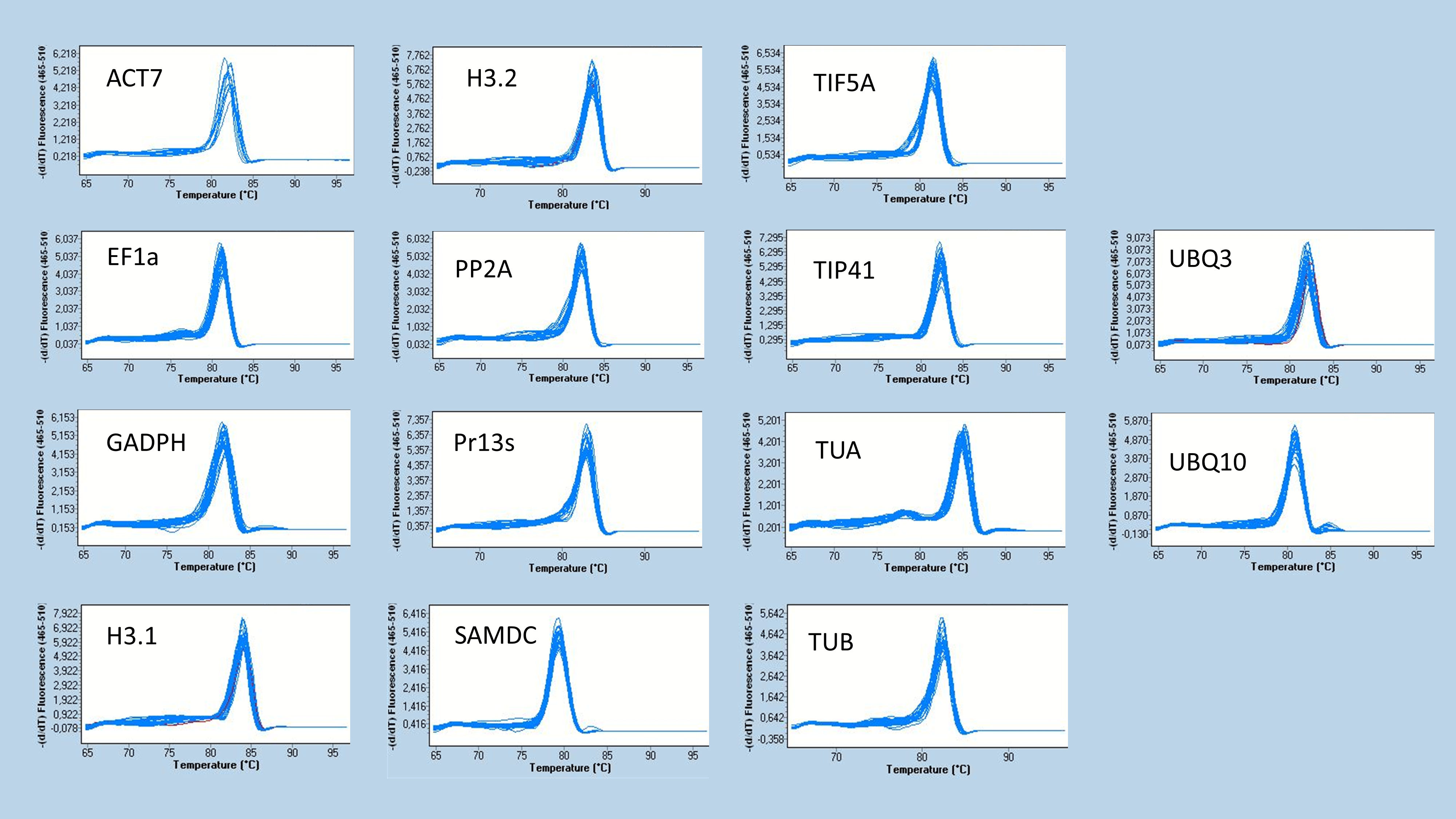

Supplement: Supplementary file 1 [file plants-11-00518-s001.zip › figS1_melting_curves.tif]

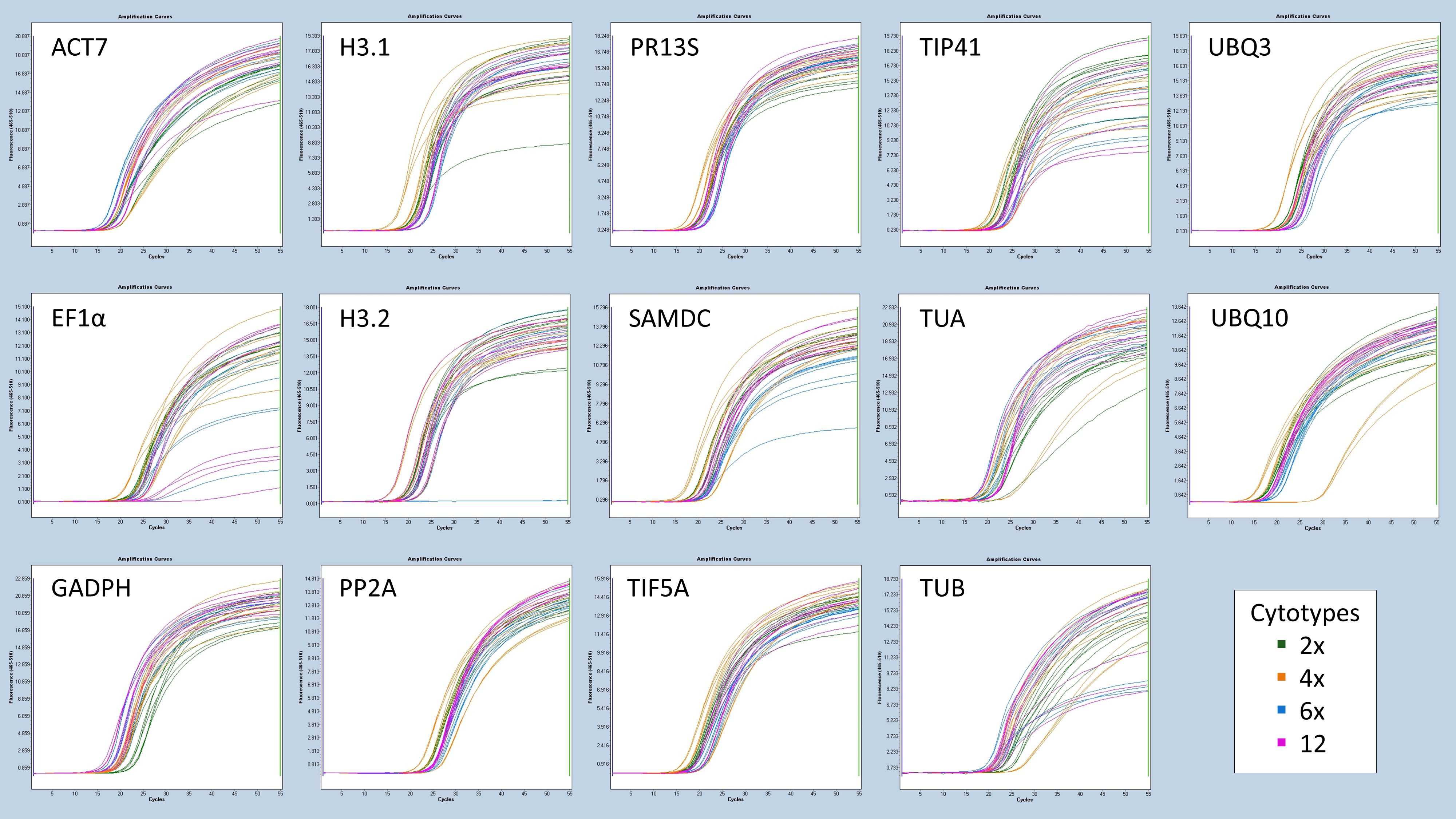

Supplement: Supplementary file 1 [file plants-11-00518-s001.zip › figS2_leafs_curves.tif]

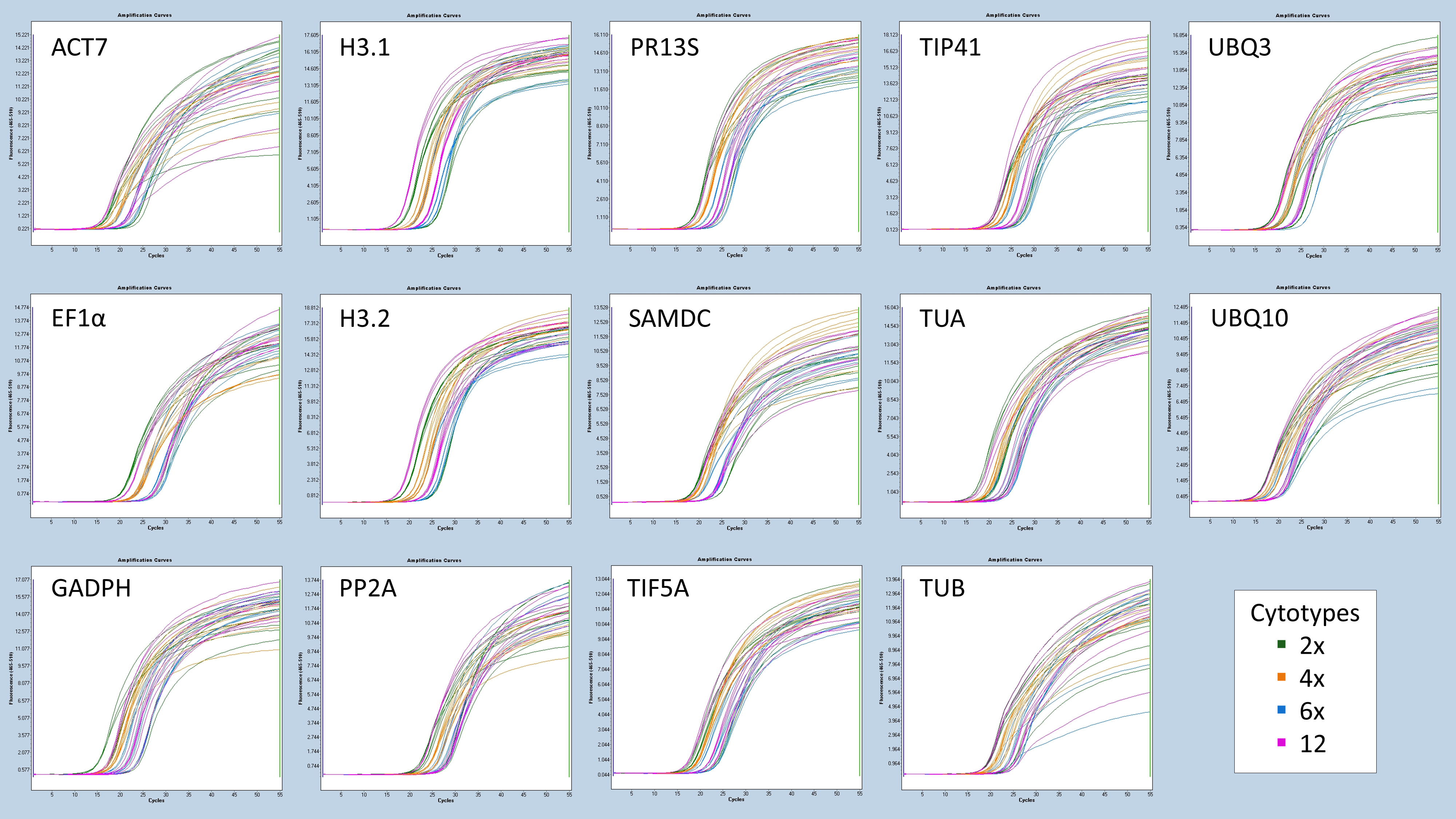

Supplement: Supplementary file 1 [file plants-11-00518-s001.zip › figS3_petal_curves.tif]
